# Heart-Type Fatty Acid-Binding Protein (H-FABP) and Its Role as a Biomarker in Heart Failure: What Do We Know So Far?

**DOI:** 10.3390/jcm9010164

**Published:** 2020-01-07

**Authors:** Richard Rezar, Peter Jirak, Martha Gschwandtner, Rupert Derler, Thomas K. Felder, Michael Haslinger, Kristen Kopp, Clemens Seelmaier, Christina Granitz, Uta C. Hoppe, Michael Lichtenauer

**Affiliations:** 1 Clinic of Internal Medicine II, Department of Cardiology, Paracelsus Medical University of Salzburg, 5020 Salzburg, Austria; r.rezar@salk.at (R.R.); p.jirak@salk.at (P.J.); mi.haslinger@salk.at (M.H.); c.seelmaier@salk.at (C.S.); c.granitz@salk.at (C.G.); u.hoppe@salk.at (U.C.H.); 2 Kennedy Institute of Rheumatology, University of Oxford, Oxford OX3 7FY, UK; martha.gschwandtner@kennedy.ox.ac.uk; 3 Institute of Pharmaceutical Sciences, University of Graz, 8020 Graz, Austria; rupert.derler@hotmail.com; 4 Department of Laboratory Medicine, Paracelsus Medical University of Salzburg, 5020 Salzburg, Austria; t.felder@salk.at

**Keywords:** H-FABP, heart-type fatty acid-binding protein, FABP3, fatty acid-binding protein 3, heart failure, HF, cardiac biomarkers

## Abstract

Background: Heart failure (HF) remains one of the leading causes of death to date despite extensive research funding. Various studies are conducted every year in an attempt to improve diagnostic accuracy and therapy monitoring. The small cytoplasmic heart-type fatty acid-binding protein (H-FABP) has been studied in a variety of disease entities. Here, we provide a review of the available literature on H-FABP and its possible applications in HF. Methods: Literature research using PubMed Central was conducted. To select possible studies for inclusion, the authors screened all available studies by title and, if suitable, by abstract. Relevant manuscripts were read in full text. Results: In total, 23 studies regarding H-FABP in HF were included in this review. Conclusion: While, algorithms already exist in the area of risk stratification for acute pulmonary embolism, there is still no consensus for the routine use of H-FABP in daily clinical practice in HF. At present, the strongest evidence exists for risk evaluation of adverse cardiac events. Other future applications of H-FABP may include early detection of ischemia, worsening of renal failure, and long-term treatment planning.

## 1. Introduction

According to the Global Burden of Disease study, cardiovascular (CV) diseases represent the leading cause of death among non-communicable diseases, accounting for approximately 17.9 million deaths worldwide in 2015 [1]. As described in the meta-analysis by Van Riet et al., the prevalence of all-type heart failure (HF) in the older cohort of patients (>60 years) is 11.8% [2]. Additionally, health care costs, related to HF, represent a serious economic burden to healthcare systems. Heidenreich and colleagues estimated that the total medical costs of HF in the US will increase from $31 billion in 2012 to at least $70 billion in 2030 [3]. Thus, it is not only important to find new therapeutic approaches, but also to diagnose affected individuals early and monitor therapies properly. Biomarkers for HF are subject of current research and may have the potential to, not only reduce costs, but also extend symptom-free intervals through effective therapy control.

Described for the first time in 1972, a group of cytoplasmic proteins called fatty acid-binding proteins (FABPs) has been under investigation in the scientific community [4]. To date, several subtypes of FABPs, occurring in various organ systems in different concentrations, have been discovered. These low-molecular-weight proteins (about 15 kD [5]) have been widely discussed, especially given the association of H-FABP as an independent risk factor for all-cause mortality and cardiovascular (CV) death [6]. According to the HUGO Gene Nomenclature Committee, the FABP family consists of 16 members, each encoded by a distinct gene. The probably best-known members include L- (liver), I- (intestinal), H- (muscle/heart), A- (adipocyte), E- (epidermal), Il- (ileal), B- (brain), M- (myelin), and T-FABP (testis) [7]. FABPs are involved in cellular fatty acid metabolism as they reversibly bind and transport long-chain polyunsaturated fatty acids (PUFA) from cell membranes to the mitochondria. Additionally, they contribute to cellular growth and proliferation processes, and can activate peroxisome proliferator activated receptors (PPARs). Therefore, they play a functional role in lipid metabolism and energy homeostasis [8,9,10].

The heart-type FABP (H-FABP), also known as mammary-derived growth inhibitor, is probably the best-known member of the FABP family. H-FABP is encoded by the FABP3 gene located on the 1p33-p32 region of chromosome 1 [11], whereas, RXRa, KLF15, CREB, and Sp1 were identified as transcriptional factor binding sites for different PPARs in animal studies [12]. It is expressed in tissues with high demand of fatty-acids, such as heart, skeletal-muscle, brain, kidney, adrenal gland, and mammary gland tissues, as well as in blastocysts [8]. FABP3 was also found to be expressed in γ-aminobutyric acid (GABA)-ergic inhibitory interneurons of the male anterior cingulate cortex in mice, suggesting that it has an important role also in cerebral PUFA-homeostasis [13]. H-FABP itself is abundant in the cytoplasm of striated muscle cells and is rapidly released in response to cardiac injury [14]. H-FABP is expressed more abundantly in the heart’s ventricles (0.46 mg/g wet weight) and atria (0.25 mg/g wet weight) than in skeletal muscles (e.g., the diaphragm contains 25% of the heart’s H-FABP concentration) or in other organs (less than 10% of the H-FABP content of the heart) [15]. In healthy individuals, serum levels of H-FABP are in the single digit ng/ml range [16,17,18]. Expression of H-FABP is regulated by the microRNA miR-1, which might play a role in the progression of HF itself [19]. Upon myocardial injury, H-FABP is rapidly released from myocytes into the systemic circulation, due to its small size and free cytoplasmic localization. Also, transient increases in sarcolemmal membrane permeability are suspected to permit H-FABP leakage into the systemic circulation [20,21]. This so-called “wounding” of myocytes was observed, even after short-term ventricular stress, and it may play an important role in diverse auto- and paracrine mechanisms in the pathogenesis of HF [20]. The elimination of H-FABP takes place via the kidney, explaining a shorter diagnostic window in patients with normal renal function [22]. Kleine et al., for example, reported that H-FABP plasma levels returned to baseline within 20 hours after the onset of symptoms in patients with acute myocardial infarction [23].

**Figure 1:**
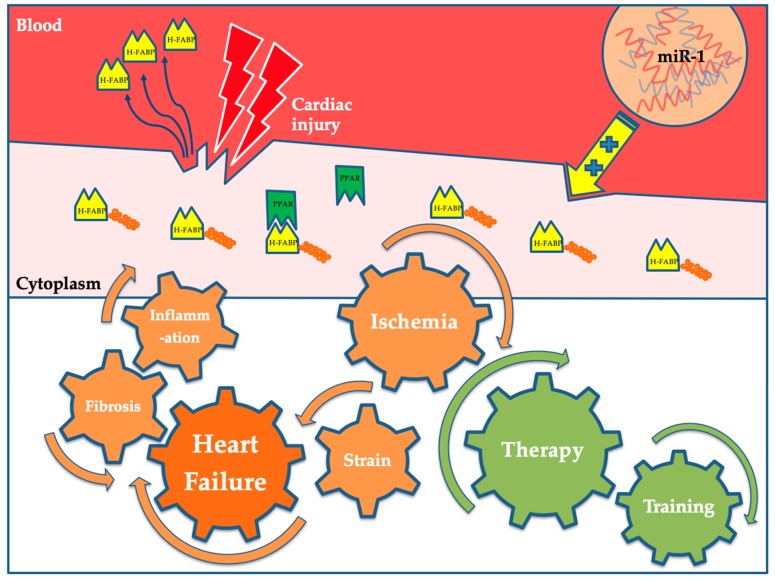
Under physiological conditions, H-FABP serves as a transport protein in cellular metabolism and can reversibly bind fatty acids. Furthermore, it can activate PPARs and therefore plays a role in lipid metabolism and energy homeostasis. The expression of H-FABP is regulated by the microRNA miR-1. In response to cardiac injury, H-FABP is rapidly released into the blood-stream where it can be quantified. Physical training as well as pharmacological interventions like anti-tachycardic therapy were shown to decrease plasma levels of H-FABP. Abbreviations: miR-1: microRNA 1; PPAR: peroxisome proliferator activated receptor (PPAR). H-FABP: heart-type fatty acid-binding protein.

Apart from its crucial role in cardiac lipid transport [24,25], several in vitro and in vivo studies investigated further functions of H-FABP. The potential role of H-FABP in cardiomyocyte differentiation was suggested by Tang et al., who observed a correlation between H-FABP expression and decreased cell proliferation in mouse cardiomyocytes [26]. A similar finding was obtained by Wang et al., using human bone marrow derived mesenchymal stem cells, by which overexpression of H-FABP inhibited proliferation [27]. Additionally, it was shown by Zhu et al., using a P19 embryonic myocardial cell line overexpressing H-FABP, that it might inhibit cell proliferation and promote apoptosis during myocardial cell development [28]. However, in a later study, H-FABP silencing instead of overexpression led to reduced proliferation and increased apoptosis in the same cell line [29]. In zebrafish, the knock-down of H-FABP resulted in impaired heart development and augmented apoptosis [30,31]. In neonatal rats, H-FABP downregulation repressed cell apoptosis and improved structural remodeling in ventricular myocytes under hypoxia. On the other hand, H-FABP upregulation enhanced phosphorylation of the MAPK signalling pathway and decreased phosphorylated protein kinase B (Akt) levels, increasing apoptosis and remodeling [32]. An anti-apoptotic role of H-FABP was also found in hypoxia/reoxygenation induced H9c2 cardiomyocytes [33]. Consistent with this, H-FABP enhanced survival in human bone marrow derived mesenchymal stem cells in hypoxia [27]. Overexpression of H-FABP promoted growth and migration in human aortic smooth muscle cells [34]. In summary, the precise mechanism by which this protein influences cardiomyocyte proliferation and apoptosis remains elusive and further research is needed to explain its mode of action. Figure 1 provides a graphic overview of H-FABP under physiological and pathophysiological conditions.

Regarding laboratory testing, different types of assays are frequently used in research and clinical settings for the detection and quantification of H-FABP in serum, plasma, or whole blood. These assays comprise enzyme-linked immunosorbent assays (ELISA) [6,15,35,36,37], immunoturbidimetric assays [38,39], multiplex assays [40,41], and immunochromatographic assays [42,43]. Test times depend on the type of assay, and vary between 5 and 120 minutes (as reviewed in [44]). The varying characteristics of these tests allow flexibility when choosing the appropriate test for the desired readout under varying budget and time restrictions.

A number of authors have discussed the role of H-FABP in clinical routine since its discovery. The following literature review will consider H-FABP and its potential use as a biomarker in HF.

## 2. Methods

**Figure A1:**
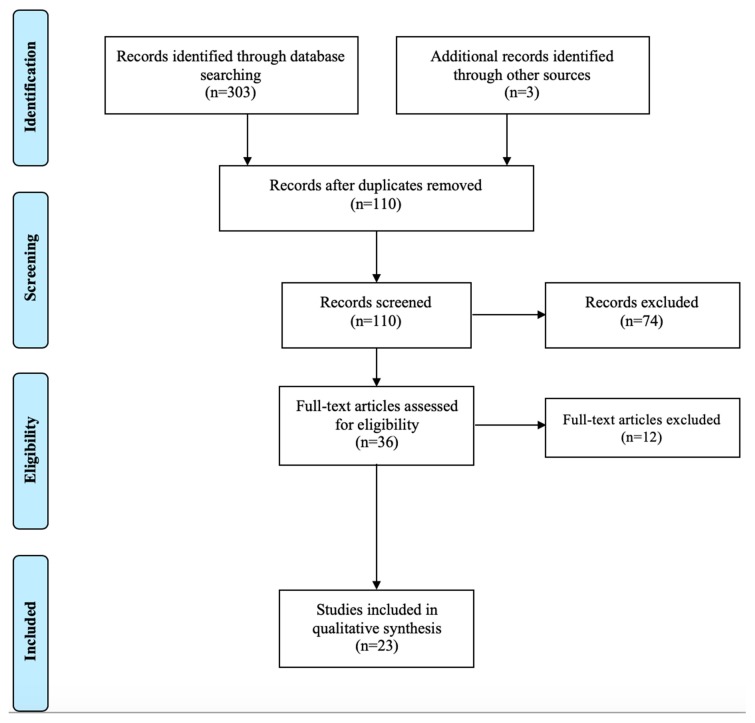
Flow diagram of the database search, screening and inclusion of the studies (modified after the PRISMA guidelines [45].

A structured database search regarding H-FABP and its role in HF was conducted using “PubMed Central”. Three researchers (R.R., M.G. and R.D.) screened the studies independently. To select possible studies for inclusion in the definite analysis, the authors screened all available studies by title and, if suitable, by abstract. Manuscripts that appeared relevant were read in full text. References of studies included were reviewed for further reading. This review on H-FABP in HF was conducted based on the Preferred Reporting Items for Systematic Reviews and Meta-Analyses (PRISMA) guidelines [45]. The corresponding flow-chart is given in Appendix A Figure A1.

## 3. H-FABP as a Biomarker in Heart Failure

According to the European Society of Cardiology (ESC) guidelines, HF is a syndrome characterised by typical symptoms and clinical signs, with a “structural and/or functional cardiac abnormality” as an underlying cause, “resulting in reduced cardiac output and/or elevated intra-cardiac pressures at rest or during stress” [46]. Due to their strong negative-predictive value, the use of natriuretic peptides is well-established in standard HF algorithms [46,47,48]. Nevertheless, like many other biomarkers, including cardiac troponins, elevated levels of B-type natriuretic peptide (BNP) may also indicate alternative conditions and BNP release may lag in conditions with very acute onset, such as flash pulmonary edema or right-sided acute HF (AHF) [46,49]. As mentioned in the actual ESC-guidelines, their use for ruling out HF, but not for setting up the diagnosis, can be recommended [46]. These guidelines also state that, despite extensive research, no recommendation can currently be made for the use of novel cardiac biomarkers in everyday clinical practice [46]. The same holds true for the American AHA guidelines on HF [47] and even a specific sub-study of the large scale PROTECT trial failed to identify the perfect single biomarker among 48 different markers for the prognostic assessment of patients with AHF [50].

Most biomarkers are not indicative of cardio-specific events but of general pathologic processes like inflammation, ischemia, fibrosis, or general cell death. As HF is an aetiologically diversified, systemic-progressive disease, a simultaneous assessment of different pathways seems reasonable, though, a prognostic assessment based on a single factor is challenging. Possible hallmarks in the pathophysiology of HF are mechanical stress, ischemia, chronic (subclinical) inflammation, fibrosis, and angiogenesis [36]. With respect to ischemic heart disease, the potential suitability of H-FABP as an early indicator of myocardial injury has been mentioned for years in numerous publications. In contrast to cardiac troponins, which are bound to the myocyte’s structural apparatus, H-FABP is present as soluble protein in the cytoplasm. Therefore, the release into systemic circulation may possibly be detected more rapidly and even after minor myocardial damage [21]. Liebetrau et al., for example, report significantly increased serum levels of H-FABP already 15 minutes after iatrogenic myocardial infarction, caused by transcoronary ablation of septal hypertrophy (TASH). in patients with hypertrophic obstructive cardiomyopathy [14]. Some authors state additional benefits of combining H-FABP with high-sensitive troponins [37,38], whereas, others do not conclude any incremental benefit of H-FABP on top of cardiac troponins for diagnosing acute myocardial infarction [42,51,52]. Regarding pulmonary embolism (PE), several publications describe the use of H-FABP for risk stratification due to its role as an early indicator of right-ventricular strain [53,54,55]. A strong correlation with the risk of major adverse events and mortality was demonstrated, and even the 2019 ESC Guidelines on the diagnosis and management of acute PE mention the use of H-FABP for risk stratification, despite the fact that prospective trials are still missing [56].

As mentioned before, H-FABP plays an important role in cellular signalling, lipid-transport, and myocytal homeostasis [57]. Additionally, due to the amphipathic nature of fatty acids, their accumulation and membranal storage can have noxious effects on cellular structural and functional properties [57]. Therefore, mechanical stress, as well as cellular damage, including from ischemic or inflammatory processes, may be further perpetuated by a disturbed myocytal homeostasis, reduced intracellular H-FABP content [11], and may support the (chronically) progressive character of HF. Despite its rapid, and in the case of CHF, sustained release into general circulation, H-FABP not only acted as an indicator of cellular damage, but also a marker of myocytal dyshomeostasis, and thus, functional impairment of the myocardium.

Various authors have investigated the role of H-FABP in patients suffering from HF with different methods over the last few years. Many studies postulate the independent relationship between H-FABP and outcome, as well as the risk of adverse CV events [40,58,59,60,61]. In a recent study by Ho et al., for example, high levels of H-FABP were an independent risk factor for CV death and acute HF-related hospitalization in 1071 patients with chronic coronary disease [40]. In an interesting study from 2005 with 186 patients, Niizeki et al. demonstrated superiority of the combined analysis of BNP and H-FABP for risk stratification in patients with CHF. The authors described the added benefit of H-FABP in showing persistent myocardial damage, compared to BNP, as a sole myocardial strain parameter. Interestingly, the authors only found a weak correlation between the two individual laboratory parameters, which may indicate different pathophysiological origins [58]. In a second study from 2008, involving 113 patients with CHF, the authors again associated persistently high levels of H-FABP with adverse events in patient follow-up. They suggested serial measurement of H-FABP concentrations for therapy monitoring, as they observed regredient serum levels under HF therapy in a subgroup of patients [59]. A significant decrease in H-FABP levels was also observed in a study by Jirak et al. where they investigated several biomarkers in fifty patients with CHF under therapy with the If channel inhibitor, ivabradine [62]. This was also observed in children with CHF after treatment with carvedilol [63].

Regarding AHF, Hoffmann et al. found improved specificity and positive predictive value for the diagnosis of AHF in their work including 401 patients with acute dyspnea or peripheral edema when using H-FABP in addition to BNP. H-FABP levels also correlated with adverse outcomes and AHF related rehospitalization [60]. These findings are in line with the work of Ishino et al. In their study on 134 patients with acute decompensated HF (ADHF), the authors were able to correlate high H-FABP levels with significantly higher rates of adverse cardiac events and in-hospital mortality [61]. Kazimierczyk et al. observed significantly higher rates of death and rehospitalization in patients with ADHF and both higher H-FABP concentrations at admission and discharge. Echocardiographic remodeling parameters correlated well with high initial H-FABP-levels [64]. Shirakabe et al. were able to correlate serum H-FABP levels not only with all-cause mortality in patients with ADHF, but also worsening of renal failure. The latter finding achieved a sensitivity and specificity of 94.7%, and 72.7%, respectively (AUC = 0.904) in non-chronic kidney disease patients [65].

Concerning patients with HF with reduced ejection fraction (HFrEF), Lichtenauer et al. enrolled 65 patients with dilative cardiomyopathy (DCM) and 59 patients with ischemic cardiomyopathy (ICM) in their study on novel cardiac biomarkers in CHF. H-FABP levels were significantly elevated in both patient populations, compared to controls without signs of HF or coronary artery disease. Furthermore, H-FABP levels not only correlated proportionally with NYHA functional class, but also inversely with ejection fraction [36]. Regarding, HF with preserved ejection fraction (HFpEF; left ventricular ejection fraction ≥50%), Kutsuzawa et al. observed an independent correlation of higher H-FABP-levels and the occurrence of adverse CV events in their study on 151 HFpEF-patients. Interestingly, serum levels of H-FABP did not differ between patients with HFpEF and HFrEF (left ventricular ejection fraction <50%) between each NYHA functional class [66]. Dinh et al. found markedly higher levels of Troponin T and H-FABP, even in patients with asymptomatic left ventricular diastolic dysfunction and patients with HF and normal ejection fraction, supposing ongoing myocytal damage in these patient collectives [67]. However, Jirak et al. observed significantly higher H-FABP serum levels in patients with DCM and ICM, than in patients with HFpEF. Nevertheless, significantly higher H-FABP concentrations were shown in HFpEF patients compared to the control group [68].

Considering patients with valvular heart disease, Iida et al. showed an independent association of H-FABP with clinical outcomes in hypertensive patients with aortic valve disease. Echocardiographically determined left ventricular dimensions were signs of cardiac remodelling and correlated significantly with measured levels of H-FABP, whereas Troponin T remained below cut-off levels in all patients [21]. Mirna et al. actually reported a significant reduction in H-FABP plasma concentration in 79 patients with severe aortic valve stenosis after conducting transcatheter aortic valve implantation (TAVI), indicating reduced ventricular wall stress and potential reversibility of cardiac remodeling due to valvular replacement [69].

Regarding arrhythmia as a co- and sometimes main-perpetrator in HF, Otaki et al. observed in their study with 402 patients higher levels of H-FABP in patients with CHF and atrial fibrillation (AF) than in patients with CHF and sinus rhythm (SR) [70]. Rader et al. showed that in 63 studied patients undergoing cardiac surgery that post- but not preoperative H-FABP levels correlated with onset of perioperative AF (POAF) [71]. Interestingly, Shingu et al. observed lower H-FABP gene expression in patients’ atria with POAF after cardiac surgery, illustrating the complexity of cellular processes in the development of HF [72].

Mirna et al. made another interesting discovery when investigating H-FABP levels in patients with pulmonary hypertension (PH). They observed that H-FABP levels were primarily elevated in group two and three PH, namely PH related to left heart disease, pulmonary disease, and chronic hypoxia. H-FABP may, therefore, be useful as a possible indicator for post-capillary PH [73].

Application of H-FABP measurement in HF monitoring may also be found in paediatric cardiology. Zoair et al. reported a correlation of serum H-FABP levels with clinical and echocardiographic signs before, and after, HF therapy in 30 children with congestive HF compared to 20 healthy individuals. An unfavourable outcome was again associated with increased serum levels. However, the study was limited as H-FABP was investigated as a single laboratory parameter, and its superiority over biomarkers, such as BNP, was not determined [74]. Sun et al. also reported that there is a correlation between H-FABP levels with disease severity in children with CHF, but again other laboratory markers were not compared [75]. In their study on 238 children and adolescents with congenital heart disease, Hayabuchi et al. found that H-FABP did not correlate with BNP, but was affected by age, NYHA class, arterial oxygen saturation, CK-MB and creatinine, supporting a different pathophysiological pathway of the two biomarkers [76]. Table 1 gives an overview of selected studies on H-FABP and HF.

## 4. Discussion and Conclusion(s)

In CV research, H-FABP represents a much-studied protein that is well-known for its role in lipid transport and influence on myocyte metabolism. Different assays and methods exist for measurement, allowing flexibility for the researcher and clinician. However, little is known about its precise function in cardiac development and remodelling. In vitro and animal studies suggest both, promoting and inhibitory roles in myocyte proliferation and apoptosis, but a mechanistic explanation is missing. If, and how, H-FABP that is released from damaged myocytes impacts the progression of HF and other CV diseases, in detail, remains unknown to date. Although, dyshomeostasis of cellular metabolism due to reduced intracellular H-FABP content, and hence, impaired fatty acid supply seems one reasonable consideration.

Individual investigators come to different conclusions about H-FABPs possible application in clinical routine. With BNP, a biomarker with high negative predictive value in differential diagnosis of HF and its long-term therapy surveillance already exists. The use of H-FABP in clinical settings has only been experimental in the past and large-scale studies are still lacking. Nevertheless, the different pathophysiological origins of H-FABP and BNP give hope for a more differentiated diagnostic approach in the future.

To date one possible application of H-FABP seems to be the detection of early and/or subclinical cardiac ischemia and inflammation. H-FABP could be used as a screening tool, for example, in routine health check-ups, since laboratory tests are inexpensive, and samples can be obtained in remote locations and analyzed in central laboratories. Takahashi et al. demonstrated a strong positive correlation between increased pulse pressure with BNP and H-FABP as signs of increased silent myocardial damage in 3504 participants at their annual health check [77]. On the other hand, the rapid detection of ischemia may pave the way for identifying patients with acute ischemia as an underlying cause of AHF at an early phase. As serum H-FABP levels were shown to correlate well with infarct size in patients with ST-elevation myocardial infarction [78], the measurement of H-FABP may enable the timely admission of revascularization procedures, and therefore, may even prevent the development of HF in the long run. As H-FABP and cardiac troponins show different release kinetics [14], a H-FABP-troponin ratio may be useful for distinguishing acute ischemia from chronic myocardial damage in patients with decompensated HF.

Furthermore, interactions of the various organ systems in decompensated HF are highlighted by several authors and international guidelines [46,65,79]. As the coexistence of HF and chronic kidney disease is frequently observed, the terms “cardiorenal syndromes” as well as “renocardiac syndromes” have gained attention in the last few years. A peculiarity of H-FABP compared to markers, such as BNP and troponins, could lie in detecting true worsening of renal function [65]. The exact mechanism that causes this correlation has not yet been clarified. High levels of H-FABP in patients with ADHF may be due to severely decompensated HF itself, but also due to damage of the distal tubules or due to accumulation in glomerular podocytes. Nevertheless, as Shirakabe et al. note, this correlation has not previously been shown for BNP or troponins, which may give H-FABP a unique position as a biomarker in HF diagnostics [65].

Another application of H-FABP as a biomarker might be in highly specialized areas. Dalos et al. observed an exponential increase of H-FABP levels, with decreasing left ventricular ejection fraction in patients with coronary artery disease, reflecting chronic myocardial ischemia [80]. As a strong and independent correlation of H-FABP with individual prognosis was shown in several studies, it may, therefore, be used in mid- to long-term treatment planning. This may be especially helpful when dealing with invasive and expensive approaches, like implantable cardiac resynchronization devices, valve replacement, or mechanical circulatory devices. For example, Cabiati et al. demonstrate an association between high H-FABP levels and poor prognosis in patients after LVAD implantation [81].

The clinical picture of HF comprises a group of heterogenous disease entities as an underlying cause. Novel biomarkers extend our understanding both of CV physiology and pathophysiologic processes, leading to cardiac remodelling and the development of HF. By defining an appropriate patient population in the right clinical context, the additional diagnostic value of H-FABP as a biomarker in HF may well be obtained in the future. Furthermore, an optimal point in time for sample recovery, as well as different thresholds for diagnostic, prognostic, and therapeutic consequences need to be determined.

We currently assume that H-FABP is, not only a rapid indicator of myocardial ischemia, but that its loss from the cardiomyocytes´ cytoplasm may cause an intracellular metabolic dyshomeostasis, and is therefore, conducive to the progressive nature of heart failure. H-FABP’s present and future in HF diagnostics may also not lie in its use as a single laboratory value, but in a combination of clinical assessment, imaging, and a multi-biomarker approach.

## Figures and Tables

**Table 1:** Overview of different positive clinical studies assessing the diagnostic value of H-FABP (heart-type fatty acid-binding protein) in patients with heart failure (HF) (sorted by main topic and year of publication).

Main Findings	Study	Patient Number	Reference
High H-FABP (>4.3 ng/mL) and elevated BNP (>200 pg/mL) showed highest rates for cardiac death and cardiac events and were also independent predictors of cardiac events (H-FABP HR 5.416, *p* = 0.0002; BNP HR 2.411, *p* = 0.0463)	Prospective study for 534+/−350 days on CHF patients	186	Niizeki T. et al., 2005 [58]
Persistently high H-FABP levels at hospital discharge (>4.3 ng/mL) correlated with increased rates for CV events (HR 5.68)	Prospective study for 624+/−299 days on patients with CHF	113	Niizeki T. et al., 2008 [59]
Two-fold higher rate of primary CV events between high H-FABP (>4.143 ng/mL) vs. low H-FABP group (32% vs. 16% respectively)	Prospective multicenter study for 24 months on patients with stable coronary heart disease (SCHD)	1071	Ho S. et al., 2018 [40]
H-FABP levels of >5.7 ng/mL were correlated with significantly higher in-hospital mortality (6.7% vs. 0%, *p* < 0.05) and cardiac events	Study for 615 days on patients with ADHF	134	Ishino M. et al., 2010 [61]
Highest H-FABP level patient quartile showed increased all-cause mortality (HR: 2.1–2.5, *p* = 0.04) and AHF related rehospitalization rate (HR 2.8–8.3, *p* = 0.001); combining H-FABP & NT-proBNP improves diagnostic specificity and PPV to rule out AHF	Prospective study for up to five years on patients with acute dyspnea or peripheral edema with or without AHF	401	Hoffmann U. et al., 2015 [60]
Significant positive correlation between H-FABP with echocardiographic parameters, death and rehospitalization	Study on patients with ADHF	77	Kazimierczyk E. et al., 2018 [64]
Serum H-FABP levels were significantly higher in patients with true worsening renal failure	Retrospective study on patients with AHF	281	Shirakabe A. et al., 2019 [65]
H-FABP levels are significantly higher in patients with DCM and ICM; ejection fraction correlates inversely with H-FABP concentrations	Study on the diagnostic value of novel cardiac biomarkers in patients with HFrEF	65 patients with DCM, 59 patients with ICM, 76 controls	Lichtenauer M. et al., 2017 [36]
Significantly higher levels of Troponin T and H-FABP in patients with asymptomatic LVDD and patients with HFnEF	Study on patients with HFnEF	49 patients with HFnEF, 51 patients with asymptomatic LVDD, 30 controls	Dinh W. et al., 2011 [67]
Higher H-FABP-levels correlated with adverse CV events; H-FABP levels did not differ between patients with HFpEF and HFrEF between each NYHA functional class	Prospective study on patients with HFpEF with a median follow-up of 694 days	151 patients with HFpEF, 162 patients with HFrEF as controls	Kutsuzawa D. et al., 2012 [66]
A greater rise in post-operative H-FABP levels is associated with AF after cardiac surgery	Prospective study on patients undergoing cardiac surgery	63	Rader F. et al., 2013 [71]
Optimal cut-off values for H-FABP as myocardial damage marker were higher in CHF patients with AF than in patients with SR (5.4 vs. 4.6 ng/mL)	Prospective study on patients with CHF and AF or CHF and SR with a median follow-up of 643/688 days	402	Otaki Y. et al., 2014 [70]
H-FABP levels correlate independently with age, NYHA-class, CK-MB, creatinine and arterial oxygen saturation	Study in children and adolescents with congenital heart disease	238	Hayabuchi Y. et al., 2011 [76]
Significant negative correlation between H-FABP levels and heart function (LVEF, CI, LVSF)	Study in pediatric patients with chronic HF	36 patients and 30 healthy controls	Sun Y.P. et al., 2013 [75]
Significant positive correlation between increased H-FABP levels and severity of HF and adverse outcome	Prospective cohort study for 3 months on pediatric patients with HF	30 patients and 20 healthy controls	Zoair A. et al., 2015 [74]

Abbreviations: ADHF: acute decompensated heart failure; AF: atrial fibrillation; AHF: acute heart failure; BNP: brain natriuretic peptide; CHF: chronic heart failure; CK-MB: muscle-brain type creatine kinase; CI: cardiac index; CV: cardiovascular; DCM: dilative cardiomyopathy; HF: heart failure; HFnEF: heart failure with normal ejection fraction; HFpEF: heart failure with preserved ejection fraction; HFrEF: heart failure with reduced ejection fraction; HR: hazard ratio; ICM: ischemic cardiomyopathy; LVDD: left ventricular diastolic dysfunction; LVEF: left ventricular ejection fraction; LVSF: left ventricular shortening fraction; NYHA: New York Heart Association; PPV: positive predictive value; SR: sinus rhythm.
